# Unraveling the Diverse Roles of Neglected Genes Containing Domains of Unknown Function (DUFs): Progress and Perspective

**DOI:** 10.3390/ijms24044187

**Published:** 2023-02-20

**Authors:** Peiyun Lv, Jinlu Wan, Chunting Zhang, Aiman Hina, G M Al Amin, Naheeda Begum, Tuanjie Zhao

**Affiliations:** 1 National Center for Soybean Improvement, Key Laboratory of Biology and Genetics and Breeding for Soybean, Ministry of Agriculture, State Key Laboratory of Crop Genetics and Germplasm Enhancement, Nanjing Agricultural University, Nanjing 210095, China; 2 Department of Botany, Jagannath University, Dhaka 1100, Bangladesh

**Keywords:** domain of unknown function (DUF), functional analysis, plant growth and development, response to stress, integrated approach and platform

## Abstract

Domain of unknown function (DUF) is a general term for many uncharacterized domains with two distinct features: relatively conservative amino acid sequence and unknown function of the domain. In the Pfam 35.0 database, 4795 (24%) gene families belong to the DUF type, yet, their functions remain to be explored. This review summarizes the characteristics of the DUF protein families and their functions in regulating plant growth and development, generating responses to biotic and abiotic stress, and other regulatory roles in plant life. Though very limited information is available about these proteins yet, by taking advantage of emerging omics and bioinformatic tools, functional studies of DUF proteins could be utilized in future molecular studies.

## 1. Introduction

Domains of unknown function, or DUFs, are categorized in the Pfam database and named using the prefix DUF, followed by a number, such as DUF1 and DUF2. The naming scheme of DUF was first introduced by Chris Ponting through the addition of DUF1 and DUF2 to the SMART database in the 1990s, which were renamed based on their featured peptides as GGDEF (PF00990) and EAL (PF00563) domains, respectively [1]. These domains have two distinct characteristics: relatively conservative amino acid sequence and protein domain with a non-characterized function [2]. While collecting information about DUFs, the same classification method was used for other Pfam families; however, no information on DUF functional characteristics was gathered [2].

With the emergence of the post-genomic era, the presence of many annotated domains and proteins with unknown functions is one of the biggest challenges. Remarkably, in the current Pfam database (Pfam 35.0, https://pfam.xfam.org/, accessed on 9 November 2022), 4795 (24%) gene families belonging to the DUF type were found in the 19,632 entries [3]. Domains of unknown function (DUFs) are widely present in animals, fungi, and plants, with key roles mostly unknown considering the number of DUFs. In bacteria, over 2700 DUFs are found and serve as a rich source of novel protein folds and functions [4]. For example, DUF1127 [5], DUF1537 [6], DUF2324 [7], DUF3233 [8], DUF1023 [9], DUF1521 [10], DUF1792 [11], and DUF 4433 [12] were found to perform a specific function. Similarly, in plants, a large group of taxa important to human development, DUFs are widely present since important roles have only been determined for some. Distinct plant species have different DUF genes that have been investigated. On the one hand, the widespread use of forward genetics techniques has driven the research on DUF genes. For example, employing fine-mapping, genome sequencing, transcriptome analysis, and comprehensive comparative sequence analysis, a recent study determined that the *LpSDUF247* gene determines the S-locus male component of the gametophytic SI system in perennial ryegrass [13]. In addition, loss-of-function mutations of DUF1668-containing genes represent the genetic events contributing to hybrid incompatibilities [14]. On the other hand, the era of big data has also brought opportunities for the functional characterization of DUF genes. In recent years, our understanding of DUFs has increased through reverse genetic approaches. In *Arabidopsis thaliana*, DUF6 [15], DUF26 [16], DUF246 [17], DUF538 [18], DUF579 [19], DUF617 [20], DUF642 [21], DUF647 [22], DUF724 [23], DUF784 [24], DUF1117 [25], DUF1218 [26], DUF4005 [27], and DUF4228 [28] have been functionally characterized. Some of them, such as DUF6, DUF246, and DUF579, are involved in cell wall development; the DUF538 protein is involved in trichome development; and DUF26, DUF1117, and DUF4228 are involved in plant stress responses. In summary, these studies revealed the involvement of DUF-domain-containing proteins in diverse biological processes.

However, the lack of genome-wide analysis of genes containing DUF domains has limited the complete understanding of their evolutionary history and biological functions. In the twenty-first century, with the advancement of science and technology, understanding the annotation and characterization of DUFs should be considered a challenge and an opportunity. To better understand DUFs in plants, we, therefore, review their functions in regulating growth and development, biotic and abiotic stress responses, technical approaches and prospects for DUFs research.

## 2. Role of DUFs in Plant Growth and Development

Domains of unknown function (DUFs) play a key role in the development of the plant. Proteins containing DUFs constitute 20 percent of all proteins [4,29]. Recently, genome sequencing and proteomics breakthroughs have made it easy to identify and sequence multiple superfamilies containing DUFs [30]. Few studies have been reported in *Arabidopsis thaliana*, *Oryza sativa* L., and other plants characterizing a few DUF families, viz., DUF1218 [26], DUF231 [31], DUF579 [32], DUF246 [17], DUF642 [33], DUF266 [34], DUF1618 [35], DUF2775 [36], DUF726 [37], DUF247 [13], DUF1668 [14], DUF538 [38], DUF827 [39], DUF724 [23], DUF1005 [40], DUF630 [41], DUF632 [41], and DUF3444 [42]. DUFs are considered to be involved in various functions related to enhancing plant growth and development (Table 1).

### 2.1. Regulation of Plant Cell Wall Development

The degradation of cellulose, pectin, hemicellulose, and lignin, all components of plant cell walls, is an important source of bioenergy. Plant cell walls play an essential part in the generation of biofuels and the transformation of sugar into ethanol for use in the industry [46,47,48]. Moreover, the plant cell wall is also involved in multiple functions, such as guarding plants against pathogen attack and helping in hormonal signaling and other physiological processes [49]. The cell wall is composed of proteins and polysaccharides. Polysaccharides such as cellulose, hemicellulose, lignin, and pectin are essential components of plant cell walls. More than 2000 genes have been estimated to be required for polysaccharide biosynthesis, assembly, and structural maintenance [50,51]. A recent study showed many DUF genes participate in polysaccharide synthesis, which affects the development of plant cell walls [34]. For instance, DUF266 proteins have been categorized as ‘not classified glycosyltransferases (GTnc)’ due to amino acid similarity with GTs. It was shown that the overexpression of *PdDUF266A* in *Populus* can increase cellulose content, reduce recalcitrance and enhance biomass production [34]. In *Arabidopsis*, three categories of proteins have been identified to alter the acetylation of cell wall polysaccharides. These proteins are named reduced wall acetylation (AtRWA), altered xyloglucan (AtAXY), and trichome birefringence (AtTBR)/TBRLIKE (AtTBL); among them, AtAXY and AtTBR/AtTBL share the conserved TBL domain and DUF231. Moreover, AXY4 acts as a xyloglucan-specific O-acetyltransferase, and the loss of function of this gene terminates xyloglucan acetylation in *Arabidopsis* [52,53]. Similarly, other research findings indicated that the *AtESK1* gene belonging to DUF231 promotes xylan’s interaction with cellulose fibrils, and when AtESK1 is combined with AtTBL29, it boosts the acetylation of xylan in *Arabidopsis* [54,55]. In addition, overexpression of a DUF231-containing protein increases O-xylan acetylation and cellulose biosynthesis in *Populus* [31]. Accordingly, these outcomes demonstrate that DUF231 family proteins are important polysaccharide modifiers and affect numerous cell wall polymers in *Arabidopsis*. Xylan is the principal hemicellulose in the secondary cell walls of eudicots and the primary and secondary cell walls of grasses and cereals. A reduction in cellulose content has been recorded in many *Arabidopsis* xylan backbone synthesis mutants. For instance, the DUF579 genes (*IRX15* and *IRX15-L*) are redundantly involved in xylan biosynthesis [32]. Later, another study showed that the loss of function of these two genes could reduce cellulose content in the *Arabidopsis* mutants [56]. Furthermore, a *Populus* DUF579 gene, *PtrDUF579-3,* negatively regulates glucuronoxylan biosynthesis and glucuronoxylan structure [57]; thus, these studies show that the DUF579 genes play diverse roles in cell wall biosynthesis. The biosynthesis and function of another structurally complex class of plant cell wall polysaccharides, pectins, are still poorly understood. To date, few enzymes involved in pectin biosynthesis have been described. For instance, one study found that a highly conserved putative glycosyltransferase-producing gene (containing the DUF246 domain) called *Pectic Arabino Galactan synthesis-Related* (*PAGR*) affects the biosynthesis of rhamnogalacturonan-I arabinogalactans and is essential for pollen tube growth [17].

In recent years, our understanding of the molecular mechanisms underlying secondary wall formation has been increased through both forward genetic approaches and genomics followed by reverse genetics. Combining *Zinnia elegans* in vitro tracheary element genomics with reverse genetics in *Arabidopsis*, a study found that WAT1 (DUF6) is a tonoplast-localized protein that functions downstream of *FRA3* and upstream of *NST1/SND1* in the signaling cascade, leading to secondary cell wall formation in fibers [15]. Few genes containing DUF1218 are reported to perform an important role in the vascular system and influence other important aspects of cell biology [58,59,60,61,62]. Moreover, previous findings have studied the expression of two genes, viz., *At4g27435* (DUF1218-encoding gene) and the *CesA* gene, and found that the cellulose content of the mutant remained unchanged compared to the control [62,63]. Additionally, another DUF1218-encoding gene (*At1g31720*) was highly expressed in the *Arabidopsis* stem and, at the same time, co-expressed in the cell wall cellulose synthase gene along with lignin- and xylan-linked genes. These outcomes showed the involvement of the *DUF1218* gene family in xylogenesis and secondary cell wall biosynthesis [26,63].

### 2.2. Role in Reproductive Development

Incompatibility during reproduction is a general phenomenon that can occur in interspecific and intraspecific hybridization and is termed hybrid incompatibility (HI). HI usually interferes with gene transfer among different species and results in stunted plant growth, hybrid sterility, and cell death deregulation [64,65,66,67,68,69,70]. Thus, for crop improvement, it is very important to understand the molecular and genetic mechanisms of HI. However, scientists are trying to discover the gene families involved in HI regulation [71]. In rice, a gene causing pollen and embryo sterility in hybrids is found at S1 and S5 [35,72], whereas the *DUF1618* gene causing sterility in male rice hybrids was mapped on Chr 12. Moreover, it was reported that loss of function in this domain could induce HI [35,73]. Another important specific tissue protein (STs) family is present in the Asteraceae and Fabaceae and consists of the DUF2775 domain [36]. This domain is considered to be linked with early fruit development, seed germination, and cell elongation [74,75,76,77]. Homomorphic SI prevents self-pollination through physiological recognition of self-pollen by the style and is a widespread mechanism occurring in over half of the angiosperms [78]. A study found that a gene encoding a DUF247 domain protein cosegregates with the S self-incompatibility locus in perennial ryegrass [13]. Loss-of-function mutations of DUF1668-containing genes represent the candidate causal genetic events contributing to hybrid incompatibilities. This indicates that DUF1668-containing genes in multiple lineages are responsible for F1 pollen sterility in rice [14].

### 2.3. Trichome Development

It is worth noting that the functions of DUF538 proteins in plants are complex and diverse. Several studies have reported the importance of plant DUF538 proteins. The DUF538 proteins are emergent plant growth regulators and usually affect phosphoinositide signaling, trichome development, and endoplasmic-reticulum-associated stress response and may function as hydrolase enzymes in plants [79,80,81,82,83]. This review mainly introduces its role in the development of trichomes. Smaller trichomes with variable branches (SVB) belongs to a terrestrial plant-specific DUF538 domain-containing gene family. It was reported that SVB-like (SVBL) and its closest relative SVB moderate trichome development and plant growth in *Arabidopsis thaliana* [18]. Although no growth defect has been recorded in the case of any single mutants, dwarfed plant growth has been reported in double mutants (*svb* and *svbl*). Meanwhile, the translational reporter assay revealed that both at the subcellular and tissue level, SVBL and SVB share highly similar localization patterns, suggesting that SVBL and SVB target a specific set of trichome development regulators and hence play a key role in trichome development and plant growth. Similarly, the transcriptomic analysis of the *gl3-sst sim* gene reported the function of the DUF538 protein in trichome development [38]. These studies imply that DUF538 proteins play important roles in plant growth and development.

### 2.4. Other Essential Growth and Development

It has been reported that some other DUF genes are associated with seedling development, root development, plant cell growth, and chloroplast movement. For example, one study identified and characterized a rolling and erect leaf mutant in rice and named it *rel2*, encoding an unknown function protein that contains DUF630 and DUF632 domains that control leaf rolling in rice [41]. Expression pattern analysis showed that *CiDUF1005* genes in *Caragana intermedia* were differentially regulated under conditions including cold, heat, dehydration, and drought treatments, as well as under the hormone abscisic acid (ABA). In addition, compared to the wild type, transgenic lines with heterologous *CiDUF1005* expression in *Arabidopsis thaliana* had longer primary roots and a greater number of lateral roots [40]. DUF827 proteins could mediate protein–protein interaction and were involved in the chloroplast photorelocation movement response [39]. The functional conservation between the DUF724 proteins and FMRP suggests that DUF724 proteins might play conserved and novel roles in RNA transportation and be involved in the polar growth of plant cells in *Arabidopsis thaliana* [23]. A mutation of the *OsSAC1* gene (containing the DUF4220 and DUF594 domains) causes sugar accumulation in rice leaves [43].

Equally importantly, the functions of some DUF genes are diverse and complex, as mentioned earlier in *DUF538* genes. For instance, it is reported that a gene containing DUF266 is involved in leaf senescence in addition to the previously mentioned role related to cell wall development [44]. The study found the DUF266-containing gene *OsPLS3* plays an important role in the onset of leaf senescence through perturbing ethylene production in leaves, thereby affecting the onset of leaf senescence in rice. In addition, the DUF1218-containing gene *VCC* plays a redundant role in early leaf margin patterning and is necessary for bilateral symmetry. Loss-of-function vcc alleles lead to unexpected changes in the size, shape, and spatial structure of the auxin and CUC2 leaf margin domains and the early loss of leaf bilateral symmetry [45].

## 3. Role of DUFs in Plant Biotic and Abiotic Stress

Numerous environmental stresses, both biotic and abiotic, are experienced by plants. However, these stresses may have a negative impact on a plant’s development, including its survival, growth, and productivity [84,85]. Few studies have shown that certain DUFs are involved in various functions related to conferring resistance to biotic and abiotic stresses (Table 2).

### 3.1. Biotic Stress

The domain of unknown function 26 (DUF26; Gnk2 or stress-antifungal domain; PF01657) is an extracellular domain in three plant proteins. The first class is CYSTEINE-RICH RECEPTOR-LIKE SECRETED PROTEINs (CRRSPs) and of which Gnk2(DUF26) from *Gingko biloba* acts as a mannose-binding lectin in vitro with antifungal activity [101,102]. In addition, another study discovered two maize CRRSPs that had also been shown to bind mannose and participate in the defense against a fungal pathogen [103]. The second class, CYSTEINE-RICH RECEPTOR-LIKE PROTEIN KINASES (CRKs), controls stress responses and development in *Arabidopsis* and rice. For example, the overexpression of the *CRK13* (an *Arabidopsis* cysteine-rich receptor-like kinase) gene results in enhanced resistance to *Pseudomonas syringae* [104]. The third category of DUF26-domain-containing proteins is the PLASMODESMATALOCALIZED PROTEINS (PDLPs), which contain two DUF26 domains in their extracellular region and a transmembrane helix but lack a kinase domain. Research studies revealed that PDLPs are linked with plasmodesmata, are involved in symplastic intercellular signaling [105], pathogen response [106], systemic signaling [107], and regulation of callose deposition [108], and act as targets for viral movement proteins [109].

Negative regulation of DUF genes has also been reported to control biotic stress responses. For example, in *Arabidopsis*, *AtDUF569* negatively regulates biotic stress responses as the resistant phenotype of the *atduf569* KO mutant may be due to the upregulation of SA-dependent PR genes during the initial phase of pathogenicity, which affects the impact of pathogenic effects, which in turn protects the mutant phenotype from late virulence and disease symptoms [86]. Surprisingly, it was reported that *AtDUF569* (*At1g69890*) positively regulates drought stress in *Arabidopsis* because the loss-of-function mutant *atduf569* showed significant sensitivity to drought stress and significantly lower abscisic acid accumulation compared with WT Col-0 plants [90].

The *At5g65040* gene (containing a DUF581 domain), named *Increased Resistance to Myzus persicae 1* (*IRM1*), was used in a study that showed that overexpression of the cloned *IRM1* gene developed an identical phenotype to the original mutant. Conversely, an *IRM1* knockout mutant promoted aphid population development compared to wild-type ones [87].

### 3.2. Abiotic Stress

Abiotic stresses, such as drought, flooding, salt stress, heat, cold, high radiation, and heavy metal toxicity, have profound effects on plant growth and survival [110]. Research findings have demonstrated that some DUF-domain-containing proteins play a vital role in plant stress responses. Some of these DUF proteins are not only involved in a single stressful environment. For example, there are a large number of really interesting new gene (RING)-domain-containing E3 ubiquitin ligases in *Arabidopsis*; among them, *At2g39720* (*AtRHC2A*), *At3g46620* (*AtRDUF1*), and *At5g59550* (*AtRDUF2*) are identified as having DUF1117 in their C-terminal regions [25]. It is suggested that the *RDUF* genes adapt biotic and abiotic plants to their environment. For example, the E3 ligase *AtRDUF1* (DUF1117) positively regulates salt-stress responses in *Arabidopsis thaliana* [93], and the suppression of *AtRDUF1* and *AtRDUF2* reduces tolerance to abscisic acid (ABA)-mediated drought stress in *Arabidopsis* [25]. In addition, researchers discovered the role of *GhRDUF4D* against *Verticillium dahliae* infection in cotton, which will help to understand the function of the *RDUF* genes in plant immunity [88].

#### 3.2.1. Drought and Salt Stress

Some DUF genes are involved in the drought- and salt-stress response. The overexpression of *GmCBSDUF3* (containing one domain of unknown function (DUF21)) could enhance tolerance to drought and salt stress in *Arabidopsis* [89]. The *AhDGR2* gene in *Amaranthus hypochondriacus* encodes a DUF642-domain-containing protein, and overexpression of AhDGR2 in transgenic *Arabidopsis* plants presents increased sensitivity to NaCl treatment [91]. The *OsDSR2* gene, which encodes a DUF966-domain-containing protein, also negatively regulates salt and simulated drought stresses and ABA signaling in rice, which provided some useful data for understanding the functional roles of *DUF966* genes in abiotic stress responses in plants [111]. Furthermore, analysis of gene expression profiling data showed that some *TaDUF966* genes were induced by salt stress in wheat (*Triticum aestivum L.*) and further confirmed the role of *TaDUF966-9B* in salt stress using virus-induced gene silencing (VIGS) assay [92]. *OsSIDP366*, a gene containing DUF1644, may function as a regulator of the PBs/SGs and positively regulates responses to drought and salt stresses in rice [94]. As an important part of landscaping, turf plays a vital role in protecting, improving, and beautifying urban environments. Therefore, it is imperative to choose high-quality salt-tolerant turfgrass suitable for landscaping in areas with saline soils. A study found that *ROPGEF7* (a DUF315 protein-coding gene) and *UFSP* (a DUF1671 protein-coding gene) might play important roles in the salt-tolerance process in *Z. japonica* and might have contrasting functions [95].

#### 3.2.2. Signaling Pathway

Several DUFs participate in regulating the signal pathway related to plant stress resistance. For example, protein–protein interaction network analysis indicated that AtDUF506s may potentially interact with iron-deficiency response proteins, salt-inducible transcription factors, or calcium sensors (calmodulins), implying that *DUF506* genes have distinct biological functions, including responses to environmental stimuli and nutrient deficiencies, and participation in Ca(2+) signaling [96]. In addition, bimolecular fluorescence complementation and calmodulin (CaM)-binding assays showed that AtRXR3(DUF506) interacted with CaM in the presence of Ca2+. Moreover, cytosolic Ca2+ ([Ca2+]cyt) oscillations in the root hairs of *rxr3* mutants exhibited high frequencies and dampened amplitudes compared to wild-type ones. Thus, AtRXR3 is a novel calmodulin-interacting protein that represses root hair elongation in *Arabidopsis* [97]. Furthermore, AtRXR3 can attenuate P-limitation-induced root hair growth through mechanisms that involve RSL4 and interaction with CaM to modulate tip-focused [Ca2+]cyt oscillations [97]. So far, the regulatory mechanism of the BES1 transcription factor has been identified and clarified in the model plants *Arabidopsis* and rice. The main biological function of BES1 is reflected in that it is an important regulator downstream of brassinosteroid signaling and plays an important role in plant stress response, growth, and development [98,99,100].

#### 3.2.3. UV-B

There are three types of UV rays, UV-A (315–400 nm), UV-B (280–315 nm), and UV-C (200–280 nm), although only UV-A and a small part of UV-B reach the Earth’s surface [112]. UV-B can cause stress or act as a developmental signal depending on its fluence levels. One study reported the involvement of DUF647 in root UV-B sensing in *Arabidopsis* early seedling development. *RUS1* (encoding a protein that contains DUF647) is an *Arabidopsis* mutant (*root UVB sensitive 1* (*rus1*)), whose primary root is hypersensitive to very low-fluence-rate (VLF) UV-B. Under standard growth-chamber fluorescent white light, *rus1* displays stunted root growth and fails to form postembryonic leaves [22].

## 4. Research Methods for DUF Proteins

The lack of genome-wide analysis of genes containing DUF domains hinders a comprehensive understanding of their evolutionary history and biological functions. Therefore, identifying the function of DUFs is extremely important for characterizing organisms. In general, there are three ways to represent the function of DUF genes. The first way is to characterize DUF genes at the structural genomics level. In recent years, structural genomics projects have propelled technology development and solved the structures of literally hundreds of proteins within uncharacterized families [2], such as DUF194 [113], DUF442 [114], DUF1110 [115], and DUF1470 [116]. The second involves using bioinformatics to identify the function. With the rapid increase in available genome sequences and structural genomics, there is a growing need for reliable computational methods to extract information about gene family architecture and evolution and thus predict the biochemical function of these proteins. This leads to a series of bioinformatics databases and tools. Here, Table 3 provides information regarding some online databases and bioinformatic tools. The third is through experimental verification. The common method to study an unknown protein’s function is to assess the consequences of a loss-of-function mutation or overexpression of the corresponding gene under multiple conditions. Generally, phenotypes can be combined with bioinformatics methods such as gene family analysis, comparative genome analysis, and evolutionary analysis to provide evidence-based annotation for some proteins. For example, a recent study identified 28 TaDUF966 proteins in wheat, and phylogenetic analysis divided these proteins into two groups. Analysis of gene expression profiling data showed that some *TaDUF966* genes were induced by salt stress. Then, virus-induced gene silencing (VIGS) assays were used to confirm the role of *TaDUF966-9B* in salt stress [92]. Additionally, genome-wide investigation and expression profiling under abiotic stresses of an unknown soybean function (DUF21) and Cystathionine-β-Synthase (CBS)-domain-containing protein family revealed tissue-specific and differential expression profiles of the GmCBSDUFs and qPCR analysis revealed that certain groups of soybean CBSDUFs are likely involved in specific stress responses. Furthermore, the overexpression of *GmCBSDUF3* could enhance tolerance to drought and salt stress in *Arabidopsis* [89].

In conclusion, with the progress of science and technology, the research technology for DUFs will become more convenient and faster, and more and more DUFs will be identified and characterized.

## 5. Conclusions and Future Prospects

To gain a comprehensive understanding of the complex mechanisms of life activity in organisms on an entirely new level, we must understand the functions of all their components. Numerous protein functions remain unknown, even in the organisms subjected to the most experimentation. For instance, 17% of the genome of the yeast *Saccharomyces cerevisiae* has not yet been characterized [117]. More than 15,537 (56%) of the 27,662 protein-coding genes in *Arabidopsis* still have uncharacterized functions in the curator summary of functional descriptions (Araport11, www.arabidopsis.org, accessed on 20 January 2023). Unfortunately, DUFs are frequently neglected due to their little relevance and are only discovered in a few genomes. In a nutshell, the present review summarizes the functional research on DUFs in plants and their regulatory functions in plant growth, development, and stress response. It is important to note that DUFs can be considered exploitable treasures given the advancement of science and technology. Although understanding the DUF genes is one of the greatest challenges in plant science, it is anticipated that a growing number of new genes with unknown functions will be uncovered and elucidated in the future. The application of computer science will be one of the benefits of studying DUF proteins; mining combined with various omics data enables researchers to predict the functional direction of proteins. The advancement of genetic and biochemical experimentation technology will speed up the comprehensive understanding of the intricate life mechanisms of the plant body.

## Figures and Tables

**Table 1:** The role of some DUFs in plant growth and development.

Plant Growth and Development	Protein (Gene_ID)	DUF (Rename)	Pfam Accession	Biological Function	Plants	Reference
Plant cell walls	WAT1 (*At1g75500*)	DUF6 (*EamA*)	PF00892	The essential role in the control of secondary cell wall formation in fibers	*Arabidopsis*	[15]
	PdDUF231A (*Potri.009G072800*)	DUF231 (*PMR5N*)	PF14416	It affects cellulose biosynthesis and plays a role in the acetylation of xylan	*Populus trichocarpa*	[31]
	PAGR (*At3g26370*)	DUF246 (*O-FucT*)	PF10250	It affects male fertility and the biosynthesis of pectic arabinogalactans	*Arabidopsis*	[17]
	PdDUF266A (*Potri.011G009500*)	DUF266 (*Branch*)	PF02485	Overexpression of *PdDUF266A* can increase cellulose content, reduce recalcitrance, and enhance biomass production	*Populus trichocarpa*	[34]
	IRX15 (*At3g50220*); IRX15-L (*At5g67210*)	DUF579 (*Polysacc_synt_4*)	PF04669	*IRX15* and *IRX15-L* affect xylan synthesis	*Arabidopsis*	[32]
	DGR1 (*At1g80240*); DGR2 (*At5g25460*)	DUF642	PF04862	L-Galactono-1,4-Lactone-responsive Genes	*Arabidopsis*	[33]
	MWL-1 (*At1g31720*); MWL-2 (*At4g19370*)	DUF1218	PF06749	*At1g31720* and *At4g19370* function redundantly to alter secondary cell wall lignin content	*Arabidopsis*	[26]
Reproductive development	LpSDUF247	DUF247	PF03140	Cosegregates with the S self-incompatibility locus	*Lolium perenne* L.	[13]
	STS1 (*LOC_Os03g02170*)	DUF726	PF05277	*STS1* is required for tapetum degeneration and pollen wall formation	*Oryza sativa*	[37]
	Sc-j (*Os03g0247300*)	DUF1618	PF07762	Loss of function in this domain could induce hybrid incompatibility	*Oryza sativa*	[35]
	S22B_j (*LOC_Os02g04420*)	DUF1668	PF07893	DUF1668-containing genes in multiple lineages are responsible for F1 pollen sterility	*Oryza sativa*	[14]
	Shoot-specific (ST) proteins	DUF2775 (*Organ_specific*)	PF10950	This domain is considered to be linked with early fruit development, seed germination, and cell elongation	Magnoliopsida	[36]
Trichome development	SVB (*At1g56580*); SVBL (*At1g09310*)	DUF538	PF04398	DUF538 affects trichome development	*Arabidopsis*	[18,38]
Other essential growth and development	MIZ1 (*At2g41660*)	DUF617	PF04759	*MIZ1* regulates lateral root development in *Arabidopsis*	*Arabidopsis*	[20]
	REL2 (*NP_001065395.1*)	DUF630; DUF632	PF04783; PF04782	Controls leaf rolling in rice	*Oryza sativa*	[41]
	DUF724 proteins	DUF724	PF05266	The DUF724 gene family is involved in the polar growth of plant cells via the transportation of RNAs	*Arabidopsis*	[23]
	WEB1 (*At2g26570*); PMI2 (*At1g66840*)	DUF827 (*WEMBL*)	PF05701	Regulate chloroplast movement velocity	*Arabidopsis*	[39]
	CiDUF1005	DUF1005	PF06219	Regulates primary root elongation	*Caragana intermedia*	[40]
	Pp1s52_118 (*Pp1s52_118V6.1*); Pp1s1_561 (*Pp1s1_561V6.2*)	DUF3444	PF11926	Role of DNA methylation in growth and differentiation in *Physcomitrella patens* and characterization of cytosine DNA methyltransferases	*Physcomitrella patens*	[42]
	OsSAC1 (*LOC_Os07G02520*)	DUF4220; DUF594	PF13968; PF04578	Causes sugar accumulation in rice leaves	*Oryza sativa*	[43]
	OsPLS3 (*LOC_Os12g42420*)	DUF266 (*Branch*)	PF02485	*OsPLS3* gene encoding a DUF266-containing protein is implicated in rice leaf senescence	*Oryza sativa*	[44]
	VCC (*At2g32280*)	DUF1218	PF06749	*VCC* is required for bilateral symmetry, but its loss of function does not visibly affect dorsoventrality	*Arabidopsis*	[45]

**Table 2:** The role of some DUFs in plant biotic and abiotic stress.

Biotic/Abiotic Stress	Protein (Gene_ID)	DUF (Rename)	Pfam Accession	Biological Function	Plants	Reference
Biotic stress	CRRSPs, PDLPs, and CRKs	DUF26 (*Stress-antifung*)	PF01657	DUF26 is land plant-specific, but structural analyses of PDLP ectodomains revealed substantial similarity to fungal lectins and thus may constitute a group of plant carbohydrate-binding proteins	Land plants	[16]
	AtDUF569 (*At1g69890*)	DUF569	PF04601	*AtDUF569* negatively regulates biotic stress responses (pathogen inoculation)	*Arabidopsis*	[86]
	IRM1 (*At5g65040*)	DUF581 (*zf-FLZ*)	PF04570	Overexpression of *IRM1* enhances resistance to aphids in *Arabidopsis thaliana*	*Arabidopsis*	[87]
	GhRDUF4D	DUF1117	PF06547	RING-DUF1117 E3 ubiquitin ligase genes in *Gossypium* discerning the role of *GhRDUF4D* in *Verticillium dahliae* resistance	*Gossypium*	[88]
Drought and salt stress	GmCBSDUF3	DUF21 (*CNNM*)	PF01595	The overexpression of *GmCBSDUF3* could enhance tolerance to drought and salt stress in *Arabidopsis*	*Arabidopsis*	[89]
	AtDUF569 (*At1g69890*)	DUF569	PF04601	Positive regulator of drought-stress response in *Arabidopsis*	*Arabidopsis*	[90]
	AhDGR2	DUF642	PF04862	Overexpression of *AhDGR2* in transgenic *Arabidopsis* plants presents increased sensitivity to NaCl treatment	*Amaranthus hypochondriacus*	[91]
	TaDUF966-9B	DUF966 (*SOK*)	PF06136	*TaDUF966* genes play a vital role in salt-stress tolerance in wheat	*Triticum aestivum*	[92]
	AtRDUF1	DUF1117	PF06547	The E3 ligase AtRDUF1 (DUF1117) positively regulates salt stress responses in *Arabidopsis thaliana*	*Arabidopsis*	[93]
	OsSIDP366 (*Os06g47860*)	DUF1644	PF07800	*OsSIDP366*, a DUF1644 gene, positively regulates responses to drought and salt stresses in rice	rice	[94]
	ROPGEF7	DUF315 (*PRONE*)	PF03759	*ROPGEF7* (a DUF 315 protein-coding gene) might play important roles in the salt tolerance process in *Z. japonica* and might have contrasting functions	*Zoysia* grass	[95]
	UFSP	DUF1671	PF07761	*UFSP* (a DUF 1671 protein-coding gene) might play important roles in the salt-tolerance process in *Z. japonica* and might have contrasting functions	*Zoysia* grass	[95]
	DUF4228 proteins	DUF4228 (*PADRE*)	PF14009	Expression profiling of the *ATDUF4228* genes under abiotic stresses (mainly osmotic, salt, and cold) and protein–protein interaction prediction suggested that some *ATDUF4228* genes may be involved in the pathways of plant resistance to abiotic stresses	*Arabidopsis*	[28]
Ca(2+) signaling	AtDUF506s	DUF506 (*PDDEXK_6*)	PF04720	DUF506 genes have distinct biological functions, including responses to environmental stimuli and nutrient deficiencies, and participate in Ca(2+) signaling	*Arabidopsis*	[96]
Phosphorus (P) stress	RXR3 (*At1g62424*)	DUF506 (*PDDEXK_6*)	PF04720	AtRXR3 is another DUF506 protein that attenuates P-limitation-induced root hair growth through mechanisms that involve RSL4 and interaction with CaM to modulate tip-focused [Ca2+]cyt oscillations	*Arabidopsis*	[97]
Brassinosteroid signaling	BES1	DUF822 (*BES1_N*)	PF05687	BES1 is an essential regulator downstream of brassinosteroid signaling and plays an important role in plant stress response, growth, and development.	*Cucumis sativus*	[98,99,100]
UV-B	RUS1 (*At3g45890*)	DUF647 (*UVB_sens_prot*)	PF04884	Role of root UV-B sensing in *Arabidopsis* early seedling development	*Arabidopsis*	[22]

**Table 3:** Bioinformatics databases and tools.

Category	Name Abbreviation	Description	URL
Integrative databases	NCBI	The National Center for Biotechnology Information advances science and health by providing access to biomedical and genomic information.	https://www.ncbi.nlm.nih.gov/ (accessed on 6 February 2023)
	Database Commons	Database Commons integrates relevant information for all collected databases and catalogs each database based on its data type, species, subjects, and location.	http://bigd.big.ac.cn/databasecommons/ (accessed on 6 February 2023)
	InterPro	InterPro provides functional analysis of proteins by classifying them into families and predicting domains and important sites.	https://www.ebi.ac.uk/interpro/ (accessed on 6 February 2023)
	Expasy	It is an extensible and integrative portal that provides access to over 160 databases and software tools developed by SIB Groups and supporting a range of life science and clinical research domains, from genomics, proteomics, and structural biology, to evolution and phylogeny, systems biology, and medical chemistry.	https://www.expasy.org/ (accessed on 6 February 2023)
	Ensembl Plants	Ensembl Genomes is developed by EMBL-EBI and powered by the Ensembl software system to analyze and visualize genomic data.	http://plants.ensembl.org/index.html (accessed on 6 February 2023)
Comparative genomic databases	Phytozome	Phytozome, the Plant Comparative Genomics portal of the Department of Energy’s Joint Genome Institute, provides JGI users and the broader plant science community a hub for accessing, visualizing, and analyzing JGI-sequenced plant genomes, as well as selected genomes and datasets that have been sequenced elsewhere.	https://phytozome-next.jgi.doe.gov/ (accessed on 6 February 2023)
	PLAZA	PLAZA is an access point for plant comparative genomics, centralizing genomic data produced by different genome-sequencing initiatives.	https://bioinformatics.psb.ugent.be/plaza/ (accessed on 6 February 2023)
Protein databases	UniProt	UniProt is the world’s leading high-quality, comprehensive, and freely accessible protein sequence and functional information resource.	https://www.uniprot.org/ (accessed on 6 February 2023)
	CATH-Gene3D	CATH is a classification of protein structures downloaded from the Protein Data Bank.	http://www.cathdb.info/ (accessed on 6 February 2023)
	SMART	SMART (a Simple Modular Architecture Research Tool) allows the identification and annotation of genetically mobile domains and the analysis of domain architectures.	http://smart.embl-heidelberg.de/ (accessed on 6 February 2023)
	PROSITE	Database of protein domains, families, and functional sites	http://prosite.expasy.org/ (accessed on 6 February 2023)
	PANTHER	The PANTHER (Protein ANalysis THrough Evolutionary Relationships) classification system was designed to classify proteins (and their genes) to facilitate high-throughput analysis. The core of PANTHER is a comprehensive, annotated “library” of gene family phylogenetic trees.	http://www.pantherdb.org/ (accessed on 6 February 2023)
	RCSB PDB	These data can be explored in the context of external annotations providing a structural view of biology.	https://www.rcsb.org/ (accessed on 6 February 2023)
	AlphaFold Protein Structure Database	AlphaFold, a state-of-the-art AI system developed by DeepMind, is able to predict protein structures with remarkable accuracy and speed computationally.	https://alphafold.ebi.ac.uk/ (accessed on 6 February 2023)
Gene expression databases	Expression Atlas	Database of differential and baseline expression	https://www.ebi.ac.uk/gxa/home (accessed on 6 February 2023)
	BAR	The Botany Array Resource: e-Northerns, expression angling, and promoter analyses	http://bar.utoronto.ca/#GeneExpressionAndProteinTools (accessed on 6 February 2023)
Bioinformatic tools	OrthoVenn2	For whole-genome orthologous gene comparisons and annotations across multiple species.	https://orthovenn2.bioinfotoolkits.net/home (accessed on 6 February 2023)
	eggNOG-mapper	eggNOG-mapper v2 is a tool for functional annotation of large sets of sequences based on fast orthology assignments using precomputed eggNOG v5.0 clusters and phylogenies.	http://eggnog-mapper.embl.de/ (accessed on 6 February 2023)
	STRING	Protein–protein interaction network functional enrichment analysis.	https://cn.string-db.org/cgi/input.pl (accessed on 6 February 2023)
	HMMER	Provides life science researchers with fast and sensitive homology searches	https://www.ebi.ac.uk/Tools/hmmer/ (accessed on 6 February 2023)
	SignalP	The SignalP server predicts the presence of signal peptides and the location of their cleavage sites in proteins from Archaea, Gram-positive Bacteria, Gram-negative Bacteria, and Eukarya.	https://services.healthtech.dtu.dk/service.php?SignalP-5.0 (accessed on 6 February 2023)
	PlantCARE	PlantCARE is a plant cis-acting regulatory element database.	http://bioinformatics.psb.ugent.be/webtools/plantcare/html/ (accessed on 6 February 2023)
	Phyre2	Phyre2 is designed around the idea that you have a protein sequence/gene and want to predict its three-dimensional (3D) structure.	http://www.sbg.bio.ic.ac.uk/phyre2/html/page.cgi?id=index (accessed on 6 February 2023)
	iTOL	iTOL is an online tool for displaying, annotating, and managing phylogenetic and other trees.	https://itol.embl.de/index.shtml (accessed on 6 February 2023)
	Evolview	Evolview is an interactive tree visualization tool designed to help researchers visualize phylogenetic trees and annotate them with additional information.	http://evolgenius.info/#/ (accessed on 6 February 2023)

## Data Availability

The datasets used and/or analyzed during the current study are available from the corresponding author on reasonable request.
